# Cultural adaptation of the savvy caregiver program for Korean Americans with limited English proficiency: a feasibility and acceptability study

**DOI:** 10.1186/s12877-022-03611-5

**Published:** 2022-11-18

**Authors:** Yuri Jang, Kenneth Hepburn, Juyoung Park, William E. Haley, Miyong T. Kim

**Affiliations:** 1 Edward R. Roybal Institute on Aging, Suzanne Dworak-Peck School of Social Work, University of Southern California, 669 West 34th Street, Los Angeles, CA 90089-0411 USA; 2 Department of Social Welfare, Ewha Womans University, Seoul, Republic of Korea; 3 Nell Hodgson Woodruff School of Nursing, Emory University, Atlanta, USA; 4 School of Aging Studies, University of South Florida, Tampa, USA; 5 School of Nursing, University of Texas at Austin, Austin, USA

**Keywords:** Evidence-based intervention, Cultural adaptation, Older immigrants, Dementia caregivers, Korean Americans, Limited English proficiency

## Abstract

**Background:**

Limited English proficiency (LEP) of dementia caregivers poses a critical barrier to these caregivers’ access to evidence-based interventions. In an effort to make such interventions available and accessible to dementia caregivers with LEP, in the present study we use Barrera and colleagues’ (2011) three-step model of cultural adaptation: (1) information gathering, (2) preliminary adaptation, and (3) full adaptation. Selecting Korean Americans as a target group and the Savvy Caregiver Program (SCP) as a target intervention, we demonstrate the sequential process of cultural adaption and report the outcomes on feasibility and acceptability.

**Methods:**

Preliminary adaptation with linguistic attunement was conducted by translating the SCP manual into Korean and certifying two lay individuals who were bilingual in English and Korean as Savvy trainers. The 6-week online SCP program was delivered by the two trainers in Korean with six to seven caregiver participants per trainer (*N* = 13). Feasibility and acceptability of the SCP for both caregiver participants and trainers were assessed using mixed methods, and their data then informed full adaptation.

**Results:**

Findings not only showed the initial efficacy of the linguistically attuned SCP but also suggested areas for further modification. Data-driven assessment yielded a list of recommended changes for full adaptation, which was reviewed by the SCP developer to ensure fidelity and by community and research partners to confirm contextual and cultural relevance.

**Conclusions:**

The adopted changes are broadly summarized as representing logistical, technical, and cultural issues. Given our refined set of educational materials and implementation guidelines, we discuss future directions for research and development.

## Background

Over the past few decades, a sizable of body of literature has documented the physical, emotional, and financial burden of dementia caregiving [1–3], and there have been continuing intervention efforts to alleviate caregiving stress and enhance caregivers’ health and well-being [4–7]. As the diversity of the U.S. population grows, inclusiveness and broad reach have become major challenges in delivering evidence-based interventions [8–10]. Because most interventions are developed and validated with samples from the non-Hispanic White English-speaking population, their use in racial/ethnic minority, non-English speaking populations requires cultural and linguistic responsiveness.

Caregivers with limited English proficiency (LEP), a term that applies to individuals who speak English less than *very well* [11], are a group prone to caregiving stress but underserved. Over 25 million Americans (9% of the U.S. population) have LEP [11]. The general pattern of social exclusion and health vulnerability in the LEP population is further exacerbated when this situation is combined with dementia caregiving [12, 13]. For the present study, we have selected Korean Americans as our target group because of their notably high LEP rate. Korean is the fourth most common language spoken by the U.S. LEP population [11, 14], and Korean Americans generally lack access to resources and services [15, 16].

Under the valuations of familism and filial piety [17, 18], family caregiving has been highly emphasized in Asian cultures. The patriarchal family structure and gendered role embedded in Asian societies often expect women (e.g., wives, daughters, or daughters-in-law) to assume the caregiver role [19, 20]. Although dementia caregiving in the context of family obligation and personal sacrifice is stressful, many Asian American caregivers have been excluded from the extant evidence-based caregiver interventions due to their stiff language barriers. Thus, using Korean Americans as a target group, we demonstrate a way to make an evidence-based dementia caregiver intervention available and accessible to caregivers with LEP. In the present study, the intervention chosen for adaptation is the Savvy Caregiver Program (SCP).

The SCP, an evidence-based dementia caregiver psychoeducational program grounded in social cognitive theory [21], postulates that caregiving entails the assumption of a role that is somewhat clinical and for which few caregivers have been trained [22]. It targets the enhancement of caregiver self-efficacy as a mechanism to promote effective coping responses to day-to-day caregiving challenges, in order to achieve better caregiver and caregiving outcomes. Delivered by Savvy-certified trainers via in-person or online group sessions, the SCP combines instruction, modeling, active learning, and coaching. The SCP has been found effective in improving caregiving skills, knowledge, confidence, and emotional well-being in diverse groups of dementia caregivers [23–25]. However, although many community-based organizations have adopted the SCP and implemented it satisfactorily in community settings, the program’s benefits have rarely been shared with non–English-speaking ethnic minorities [23]. Given that Korean culture places a high emphasis on teaching and learning, the SCP’s problem-focused, goal-oriented psychoeducational approach is surely relevant to our target group [26, 27]. Also, the SPC’s core value of self-efficacy aligns well with a sense of mastery, a psychological asset important among Korean Americans [28, 29].

In the present context, cultural adaptation is the “systematic modification of an intervention protocol to consider language, culture, and context in such a way that it is compatible with the client’s cultural patterns, meanings, and values” [30]. It has been highly endorsed as a method to close the language–culture gap and achieve the ideal of “equal treatment for all” [8–10]. However, cultural adaptation has been challenged by the tension between fidelity and fit [30–32]. Ensuring scientific integrity of evidence-based interventions on the one hand and responding to the unique characteristics, norms, and values of a target group on the other present a balancing act that requires a thorough understanding of both the theoretical underpinnings of interventions and the cultural contexts of target groups.

The science of cultural adaptation has been most advanced in mental health interventions [30–32]; however, in the field of dementia caregiving, the effort to tailor caregiver interventions for a particular cultural or ethnic group has been limited. According to a review by Napoles and colleagues [33], there are only 18 psychosocial support interventions that includes African American, Latino, and Asian family caregivers, and 11 of them address cultural elements in their design. It is notable that 8 of them are from the Resources for Enhancing Alzheimer’s Caregiver Health (REACH) initiative [34, 35], which made an early effort to characterize ways to culturally adapt caregiver interventions for Hispanic and African-American caregivers [4]. One example of the REACH program adaption for other groups was with Chinese immigrants in the United States, and it considered the need to provide service in the home and language differences [36]. In a more recent systematic review of dementia family caregivers in global Chinese communities [37], there were two additional studies conducted in the United States. These review studies demonstrate that there are only a few dementia caregiver interventions targeted to Asian American communities and that the extent to which these interventions address cultural issues is minimal.

**Fig. 1:**
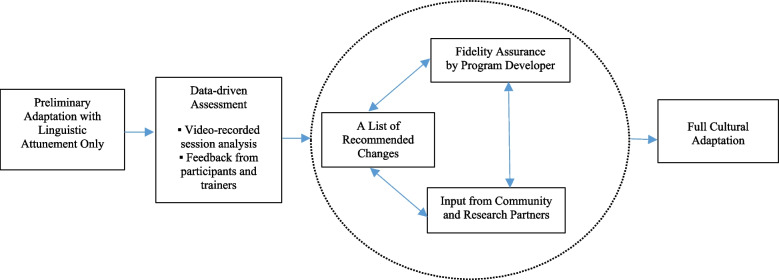
The Process of Cultural Adaptation of an Evidence-based Intervention

As a feasibility and acceptability study, the overall approach is guided by Barrera and colleagues’ [31] three-step model of cultural adaptation: (1) information gathering, (2) preliminary adaptation, and (3) full adaptation. Information gathering from the literature resulted in the informed selection of our target group, Korean American dementia caregivers with LEP, and our target intervention, SCP. The next step consisted of preliminary adaptation with surface-level language attunement, followed by full adaptation with deep structure-level modifications. This sequence is based on the notion that the effort to make an evidence-based intervention accessible to and efficacious for individuals with LEP requires cultural responsiveness that goes beyond language accommodation. The model calls for fundamental modifications that address cultural, social, environmental, and psychological characteristics of the target group [30–32]. With this sequential design, the input of caregiver participants and trainers from our preliminary adaptation testing informed our full adaptation. We employed several fidelity–fit balancing strategies suggested in the literature, such as rigorous evaluation and testing, data-driven decision-making, incorporation of input from community and research partners, and a feedback loop [38–40]. Furthermore, we included an original developer of the Savvy intervention (K.H.) to ensure that the scientific integrity of the program’s core principles and mechanisms were not compromised by our modifications. The steps of cultural modification are depicted in Fig. 1, and here we document lessons learned from each step.

## Methods

### Preliminary adaptation design

Preliminary adaptation was launched with translation of the original SCP manual for caregivers into Korean. Two individuals proficient in English and Korean undertook initial translation, and the draft was reviewed and refined by two other bilingual individuals. Using a community health worker model [41, 42], we recruited two college-educated lay individuals who were bilingual in English and Korean to serve as Savvy trainers. Both of them were middle-aged women with work experience in human services (one at a childcare center and the other at a county office). Neither of them had formal training on dementia or personal caregiving experience. To become certified Savvy trainers, they received training via an online program developed by the SCP authors and delivered by the Emory University Nursing Experience. This training provided an understanding of the SCP’s core principles and training mechanism as well as preparation for conducting SCP group sessions [23]. During program implementation, they were assisted by weekly pre-session preparation and post-session debriefing meetings with the Project Lead (YJ). During the sessions, they received technical assistance from research staff.

### Preliminary adaptation testing

Recruitment for the online psychoeducational program was conducted in the greater Los Angeles area. Eligible participants were individuals who self-identified as Korean American, were caregivers of a family member with dementia, spoke English less than *very well*, and scored lower than 20 on the Patient Health Queationnaire-9 (PHQ-9) [43]. Exclusion of those with severe depression (PHQ-9 score ≥ 20) was necessary because the SCP is not a mental health treatment program. A list of local mental health service providers was also prepared; however, no case of severe depression was identified at screening.

Given the recruitment challenge of dementia-related research in ethnic minority communities, we used a variety of sources (e.g., established community networks, referrals, and public advertisement). The project team has been conducting community-based research with the local Korean American community, including the development of area ethnic resource database and surveys/focus groups/in-depth interviews with community members. We originally intended to recruit 12 participants (six participants in each trainer’s class), but we ended up with 13. During February and March, 2022, each trainer delivered the linguistically attuned SCP to six or seven participants in Korean. This online psychoeducational program took place via Zoom on Saturdays for 6 weeks (one trainer’s class in the morning and the other’s in the afternoon), and each session lasted 75 min. The project was approved by the Institutional Review Board at the University of Southern California. All procedures were performed in accordance with the ethical principles of the Helsinki declaration. Informed consents were obtained from all participants.

Prior to program implementation, the Project Lead met individually with each participant via Zoom to explain the project, offer an opportunity to ask questions, and obtain consents. All participants were informed that the session would be observed by research staff and recorded for research purposes. During the meeting, sociodemographic characteristics and caregiving context were assessed. The meeting also prepared participants for the use of Zoom, with a brief tutorial on basic Zoom functions. Participants received weekly reminders for sessions via email along with course materials.

Upon completion of each session, research staff sent an email to participants and trainers to solicit a brief session evaluation. Using the Savvy performance checklist [22], fidelity was ensured in session observations and trainers’ self-evaluations. After completing the 6-week program, research staff met individually with each participant and trainer to obtain their feedback. Participants and trainers were asked to comment on various aspects of the program and respond to survey items intended to assess the program’s feasibility and initial efficacy. The number of sessions completed was counted, and participants’ satisfaction was measured with the Client Satisfaction Questionnaire-8 (CSQ-8) [44]. The CSQ-8 includes questions asking participants to rate the quality of service that they received, the extent that the program met their needs, and their willingness to recommend the program to others on a four-point scale. Total scores could range from 0 to 24, with higher scores indicating greater levels of satisfaction.

For initial efficacy, perceived change in each domain of knowledge, skills, attitude, and control after attending the SCP was measured with a single question; responses ranged from 0 (*very much lowered*) to 5 (*very much improved*). For trainers, the Implementation Outcome Measure [45] was used to assess the program’s perceived acceptability, appropriateness, and feasibility. Designed as a feasibility and acceptability study with a small sample, our analyses of the quantitative data were descriptive. The qualitative data enabled us to contextualize participants’ responses to the program and identify areas for improvement. The individual interviews with the 13 caregiver participants and two trainers were recorded; they were then reviewed and summarized.

### Full adaptation design

As Fig. 1 shows, full adaptation was informed by the quantitative and qualitative data generated in the preliminary adaptation testing. After assessing the data, we made a list of recommended changes for full cultural adaptation. The list was reviewed by the SCP developer (K.H.) to ensure fidelity. We also sought input from community and research partners. Five social service providers in Korean communities and six University-affiliated researchers in the field of aging, cultures, and health reviewed the list and provided their perspectives. Through this iterative process with various stakeholders, we determined areas for modification.

## Results

### Characteristics of the preliminary adaptation testing participants

The sample included 13 caregivers; their descriptive characteristics are summarized in Table 1. The mean age was 54.2 years (*SD* = 12.5; range, 31–70). Approximately 85% were women, over 15% were not married, about three quarters had more than a high school education, and 54% were employed. All participants were born in Korea, spoke Korean as their primary language, and spoke English less than *very well*—either *well* (69.2%) or *not well* (30.8%). Years lived in the U.S. averaged 27.1 (*SD* = 7.93; range, 18–44). About half of the participants were caring for their own parent, followed by parent-in-law (30.8%), other relative (15.4%), and grandparent (7.7%). There was no spousal caregiver. Yeas of caregiving ranged from 2 to 7, with a mean of 3.75. Care recipients’ age averaged 87.7 years (range, 66–101), and a majority of care recipients (92.3%) were women.

**Table 1:** Descriptive Summary of Participating Caregivers

Class	ID	Age	Gender	Marital Status	Relation to Patient	Age of Patient	Years of Caregiving
1	11	68	Female	Married	Daughter	97	5
1	12	53	Female	Married	Daughter-in-law	85	14
1	13	55	Female	Married	Niece	72	10
1	14	32	Female	Single	Granddaughter	85	3
1	15	59	Female	Married	Daughter	95	4
1	16	60	Female	Married	Niece	87	1
2	21	59	Female	Single	Daughter	89	3
2	22	70	Male	Married	Son	96	10
2	23	58	Female	Married	Daughter	92	3
2	24	63	Male	Married	Son	90	15
2	25	39	Female	Married	Daughter-in-law	66	3
2	26	58	Female	Married	Daughter	84	5
2	27	31	Female	Married	Daughter-in-law	100	4

### Evaluation of the preliminary adaptation

The quantitative data demonstrated positive feasibility and initial efficacy of the preliminary adaptation. The number of completed sessions averaged 5.92 (*SD* = 0.27); over the 6-week period, there was only one absence. The session completion rate was 98% (one absence out of a total of 78 sessions; 6 sessions × 13 participants). Participants’ satisfaction scores were high, with a mean of 31.1 (*SD* = 1.16; range, 29–32). All participants reported either ‘much’ or ‘very much’ improvement in all areas of knowledge, skills, attitudes, and control. Trainers’ perceived acceptability, appropriateness, and feasibility of the program were also favorable, with respective means of 16 (*SD* = 0), 16 (*SD* = 0), and 15.5 (*SD* = 0.71), out of a potential range from 4 to 20.

In the qualitative interviews, participants indicated their strong endorsement of the SCP. They appreciated the opportunity to learn and share. Noted changes credited to the intervention included understanding the new concepts of “personhood” and “contented involvement”; realizing the importance of “self-care”; an increased ability to assess situations and use appropriate strategies; help with setting short-term and long-term goals and expectations; and positive changes in outlooks, attitudes, and moods. One participant stated that she gained courage to talk about her dementia caregiving situation with her friends. Two participants noted that the program enabled them to realize that there were therapeutic benefits to talking about their emotions, and they said that they were now interested in receiving mental health counseling. All participants mentioned the online education’s convenience and indicated high satisfaction, although three participants did state a preference for in-person sessions. Nevertheless, for many participants, online education was the only feasible option for participation due to absence of other family members who could be with the care recipient during the sessions. Trainers also endorsed the SCP strongly and noted that the SCP’s principles were well received by participants. They stated that participants were highly motivated to learn new knowledge and apply it in caregiving. The trainers felt rewarded by the participants’ positive changes and appreciation of the program.

### Full adaptation design

Analysis of the mixed-methods data from our preliminary adaptation testing yielded the recommended changes summarized in Table 2. The comments are organized into three major areas: logistical, technical, and cultural issues. The table shows how comments from participants and trainers informed the recommended actions and how those actions were accepted or rejected in the feedback loop. As illustrated in Fig. 1, decisions were made in collaboration by the research team, the SCP developer, and community and research partners, with consideration of the SCP’s integrity and content/context relevance. For example, the comment that the Savvy manual was too long led to the recommendation of a summary version of the manual. However, this was rejected because of a concern about losing the integrity of the SCP given the selection of content for the summary. Instead, we decided to include an outline for each chapter of the manual in a weekly handout, so that those who were interested in specific topics could easily locate corresponding content in the manual. In addition, a suggestion to make the recorded sessions available for those who were absent was denied, in order to protect participants’ privacy.

**Table 2:** Feedback from the Caregivers and Trainers Participated in the Preliminary Adaptation Testing and Recommended Changes

Domain/Theme	Comment from Caregivers	Comment from Trainers	Recommended Change
**Logistical issues**			
Program name	Participants noted that the word “savvy” is a bit unfamiliar but fits well with the program.	Trainers felt that the program name was well-received, but they were not sure that all participants had fully appreciated the intention behind it. The word “savvy” had a powerful impact.	• To keep the English word “Savvy” without translation: Savvy 부양인 프로그램 • To include a brief discussion of the program’s name during the first session • To brand the program as “K-Savvy”
Session frequency and length	There was consensus that the frequency of 6 weekly sessions was relevant but that the session length of 75 min was rather short. Participants expressed their strong desire to have more time for discussion.	Trainers noted their challenges with time management. In some cases, they had to stop meaningful discussions to cover the course materials allocated for the week. During the session, all participants seemed to stay focused, and no fatigue was observed. Trainers also recommended adding 10–15 min to each session.	• To keep the frequency of 6 weekly sessions • To increase the session length to 90 min
Class size and composition	A majority reported that the six or seven participants per session were adequate. One participant stated that adding one or two more would be beneficial for generating more diverse conversations. Participants indicated that they felt more comfortable when there were other members who shared similar characteristics.	Trainers stated that a bigger class size than six or seven participants would be hard to manage. Trainers were concerned that each participant would then have even less time to share their opinions. Trainers also noticed diversity among participants (e.g., age, gender, relation to care recipient, dementia severity of care recipient) and made efforts to make all participants feel included.	• To limit the number of participants to six or seven • To consider participants’ characteristics in class assignment • To prepare trainers to better attend to diversity among class participants
Accommodation for schedule conflict	One participant in the afternoon class had to miss Session 4 due to a schedule conflict. Another person in the afternoon session who had to miss Session 5 volunteered to attend the morning session. She enjoyed attending the alternative class and interacting with new people. There was another case of cross-group participation in the Session 6.	In Session 5, the trainer in the morning session noted that having a guest from the afternoon class brought new energy to the class. The trainer in the afternoon session stated that the smaller class size than usual made the participants more engaged in conversations, and she felt more control over the class.	• To inform participants of the option of attending an alternative class in case of schedule conflict (Not accepted, owing to concerns about protection of participants’ privacy)
Manual	In general, the quality of Korean translation of the Savvy manual was rated high, but there were parts to be revisited. With regard to the length of the manual (290 pages), participants’ response was mixed. Some participants demonstrated deep appreciation of the detailed information, but others reported that they were overwhelmed by its volume and could not find time to read the entire document.	Trainers stated that they were very impressed that many participants read the manual carefully and were well prepared for the sessions. They also noted the value of the manual’s extensive content, because they were not able to cover all of the information during the limited session time. Trainers strongly endorsed the value of the manual as a reference.	• To refine and finalize the K-Savvy manual (Not accepted, in order to maintain the program’s integrity)
Trainer training	Participants made favorable comments on the trainers (e.g., knowledgeable, teaching/listening well, responsive, calm). Some participants were interested in learning about the Savvy trainer certification.	Trainers expressed initial discomfort in teaching participants who were older and had everyday caregiving experience. They noted that the pre-session preparation and post-session debriefing were very helpful. They also indicated a need for more training, particularly on coaching skills.	• To develop an introductory video clip, including endorsement of Savvy trainers by the SCP developer (K.H) and the Project Lead (Y.J.) • To enhance the pre-session prep/post-session debriefing sessions for trainers • To consider developing a booster training session for the K-Savvy trainers
Formal acknowledgement of achievement	In the closing session, participants enjoyed sharing their experiences and changes over the past 6 weeks. They expressed regret for not participating in person. One said that she felt awkward to exit the Zoom meeting without properly thanking the trainer and other participants.	Trainers noted that they became quite emotional during the last session, with the feeling that they had built a strong bond with participants. They had given much thought to their final remarks but still wondered what would be a proper closing. Trainers wished that they had a more formal way to recognize participants’ achievement.	• To ensure enough time for all participants to share their final remarks • To offer a Savvy Caregiver Certificate for those who complete the program
**Technical issues**			
Virtual class environment	All participants attended the sessions without any major technical difficulty. However, a few participants noted that there were times that they were bothered by an unstable Internet connection, poor sound quality, and background noise of other participants.	Trainers reported that it took some time to get used to the Zoom functions (e.g., screen share). Session technical assistance by research staff (e.g., muting participants with background noise, changing names on participants’ screens, playing video clips on behalf of the trainer) was helpful.	• To utilize the pre-meeting with participants to help them become technically prepared (e.g., equipment checking, Zoom tutorial, overview of online etiquette) • To enhance technical training for trainers • To continue in-session technical assistance
**Cultural issues**			
Cultural orientation of Savvy core principles	Participants indicated their strong endorsement of the SCP core principles. They were appreciative of learning new concepts of “personhood,” “contented involvement,” and “self-care.”	Trainers noted that discussions on personhood and self-care went very well. They noticed that several caregivers never thought of self-care and some associated with feelings of guilt.	• To reframe the seemingly individualistic SCP values in the context of collectivistic cultures
Cultural context of caregiving	Three female participants were caring for their mothers-in-law, and they noted unique challenges that put them apart from other caregivers. One participant called attention to the fact that some caregiving situations are based on obligation and duty.	Trainers noted a need to address culturally specific issues in caregiving and use various forms of caregiving situations as examples.	• To ensure ways to address participants’ unique situations and foster everyone’s sense of belonging
Special interest in family issues	Participants wanted to have more information and discussion of the caregiving resource map in Session 5 and caregiving arrangement type in the Session 6.	Trainers noted participants’ strong interest in the family-related topics and the shortage of time for enriched discussion on those topics. One trainer noted that leading discussion on a sensitive topic was challenging. She felt she was not prepared to properly respond to the comments made by participants.	• To allocate more time to cover the caregiving resource map in Session 5 and caregiving arrangement types in Session 6 • To include how to handle sensitive topics in trainer training • To develop a list of potential questions and responses for trainers
Cultures in group discussion	Some participants reported that they were a bit reluctant to speak in group discussion. A 31-year-old participant said that she usually waited out of respect until older participants were done speaking, but she often ended up not having a turn. Another participant said that he yielded opportunities to speak for others who were in a more desperate situation. One female caregiver of a patient in an early stage of dementia stated that she was quite reluctant to talk about her mother because she felt sorry for another participant whose mother was in an advanced stage.	Trainers stated that they made efforts to offer a comfortable space to share opinions. They tried to give all participants an equal opportunity to speak. The online mode was a bit challenging for reading all participants’ momentary reactions and intentions to speak. Trainers also noted their challenge in embracing diversity not only in participants but also in their care recipients. Trainers also noted that they were impressed by the fact that many participants made a great effort in doing home activities and reporting them back in class. They felt that their acknowledgement of the effort was not enough.	• To train trainers on how to facilitate discussions, make all participants engaged, and address issues of diversity • To include a brief talk on group discussion encouraging all participants’ active engagement in the first session • To set rules and expectation for class participation addressing traditional culture influenced communication style (e.g., passive communication, self-effacement, expressing empathy) • To include coaching strategies for positive reinforcement in trainer training
Cultural relevance of audiovisual materials	Participants made favorable comments on the use of audiovisual materials. The individuals featured in the video clips and photos were mostly Whites, and responses were mixed. Some participants stated that they would feel much closer if the individuals featured were Korean or other Asian individuals, while others noted that the audiovisual materials in themselves had an impact beyond the language and cultures.	Trainers felt that the audiovisual materials had a powerful impact, and they highly enjoyed leading those activities. Although the general message from the video clips seemed to be conveyed well, they were not sure that all participants had a full understanding of the detailed conversations presented in the video clips.	• To include Korean subtitles in the video clips • To replace some photos in the manual with those featuring Korean/Asian individuals
Culture-specific example	Although the examples of tasks and activities used in the class (e.g., making sandwiches, baking cookies) were generally approachable, participants enjoyed talking about comparable tasks and activities that were culturally specific.	Trainers noticed that discussions naturally led to application in the tasks and activities common in the lives of Koreans. Examples included cooking rice, trimming beansprouts/green onions, and finding matching lids for side-dish containers.	• To allocate time for culturally specific discussion in each session

## Discussion

The cultural adaptation of evidence-based interventions offers an important way to reach out to racial/ethnic minorities conventionally excluded from program delivery [8, 9, 30]. Particularly for immigrants with LEP, linguistic and cultural adaptation are indispensable for making interventions available and accessible. However, such adaption requires a rigorous process that balances fidelity and fit [30–32]. Guided by Barrera and colleagues’ [31] model of cultural adaptation, in the present study we have demonstrated the process of adapting the SCP for Korean American caregivers with LEP.

Our sequential design with preliminary adaptation (surface-level language attunement) followed by full adaptation (deep cultural adaptation) allowed us to make data-driven modifications. These steps are in line with Lau’s [40] distinction between engagement (i.e., making an intervention available and accessible to the target group) and outcomes (i.e., generating positive changes in target variables). Engaging persons who represent the population targeted by the adaptation is highly recommended [30–32], and our design offered pathways to follow such a recommendation. Findings from our mixed-methods evaluation with caregiver participants and trainers not only demonstrated positive feasibility, acceptability, and initial efficacy but also played an instrumental role in guiding full adaptation.

Despite widely known challenges to the recruitment and retention of racial/ethnic minorities in dementia-related research and clinical trials [46], we recruited more caregivers than our proposed sample size and retained all of them during our 6-week intervention. This success is attributable to our team’s ongoing engagement in building networks, partnerships, and trust in ethnic communities. In addition, the remarkable rate of session completion (98%) reflects a high need for service among these caregivers that has not been met. Satisfaction with the program was high, and all participants reported improvements in knowledge, skills, attitudes, and control after they had attended the program. The participants’ qualitative data contextualized our quantitative findings. During the interviews, many participants noted their appreciation of core values of the SCP such as personhood, contented involvement, and self-care, which suggests that the Savvy principles crossed the cultural divide and were delivered successfully. Acceptability, appropriateness, and feasibility of the program were also rated favorably by the trainers. These outcomes confirmed the relevance of the SCP for Korean Americans, whose culture places a great emphasis on learning and teaching [26, 27] and who value the sense of control as a psychological asset [28, 29]. In addition to meeting information needs (e.g., knowledge and skills gained), the SCP offered emotional benefits (e.g., a sense of community, a positive emotional state, courage to talk about personal emotions), fulfilling its psychoeducational function. As indicated by the two caregivers who were interested in receiving counseling after they participated in the SCP, exposure to the psychoeducational program seemed to foster positive attitudes toward mental health services.

The recommended changes for full adaptation were reviewed by the SCP developer for fidelity assurance and by community and research partners for contextual and cultural relevance. We exercised rigorous testing and evaluation, data-driven decision-making, solicitation of input from multiple stakeholders, and consensus building via a feedback loop to maximize the balance between fidelity and fit. The adopted changes can be summarized as logistical, technical, and cultural. First, logistical changes included a session time increase, an introductory endorsement video, branding with the inclusion of a brief discussion of the program’s name, the provision of a weekly handout with an outline of the contents in the manual, and certificates of completion. Second, technical changes included enhancement of training and provision of in-session assistance for trainers.

Finally, many of the recommendations pertained to cultural issues. It should be noted that several caregiver participants characterized SCP core principles (e.g., personhood, contented involvement, self-care) as new knowledge. Given these principles are based on individualistic values, it is necessary to reframe them in the collectivistic cultural context. For example, self-care could be introduced as a way to improve the quality of caregiving, which will benefit the care recipient and the whole family. The audiovisual materials need to be modified to be culturally and linguistically appropriate by adding Korean subtitles in video clips and replacing original photos with those featuring Korean persons. The benefit of using culturally compatible educational materials has been reported in intervention studies with diverse populations [47]. The trainers were also instructed to use culturally relevant examples in class activities and allocate sufficient time to address culturally specific issues. For example, cooking rice and trimming beansprouts were noted as being more culturally relevant activities for Korean-American older adults than making sandwiches and baking cookies. Reflecting family-oriented caregiving in Asian cultures, many participants were interested in learning about family-related topics such as mapping family resources and handling family conflicts, so we decided to enhance course coverage on such topics. It is noteworthy that two female in-law caregivers noted difficulties in their relating to the experience of other caregivers. The unique challenges of in-law caregiving have been reported in other studies with Asian Americans [19, 20]. Although the SCP acknowledges diverse patterns of family caregiving [23], culture-specific caregiving contexts need to be addressed with examples in class and relevant discussion topics. Trainers should be mindful of the diversity in caregiving relationships and the need to recognize these relationships so that all participants feel included. Also, in the sessions, younger caregivers and those caring for early-stage patients were reluctant to speak first, likely because respect for those who are older and empathy for people less fortunate are embedded in collectivistic Asian cultures [48]. Trainers should be aware of such cultural dynamics and sensitively encourage participants to take part in discussions.

The following limitations of the present study should be noted. First, our use of a small sample in one geographical location precludes generalizability. Also, our evaluation of the program’s efficacy was limited by the absence of a control condition and behavioral outcome measures. However, this was a pilot intervention project intended to explore initial efficacy, and our results were supportive. It should also be noted that there was no spousal caregiver participant in the present study, primarily due to the online delivery mode of the intervention. Given that spousal caregivers are more disadvantaged in computer usage and Internet access and have more caregiving burden than adult-child caregivers [19, 20], future studies should consider how to make the SCP online program accessible and available for the under-resourced spousal caregivers.

Despite these limitations, our findings guided the adaptation of the SCP for Korean American caregivers with LEP, resulting in a refined set of educational materials and implementation guidelines. The adapted SCP warrants a larger scale implementation to build evidence for efficacy and effectiveness. The recruitment of participants should be promoted via collaborations with health and social services and faith-based organizations in ethnic communities. Although the SCP’s principles and approaches were well-received, further attention is needed to ensure the compatibility of the values, goals, and beliefs of the SCP with those of the target group. It should also be noted that many elements of full adaptation were directed toward trainers’ awareness and management skills. Future endeavors should focus on developing a rigorous train-the-trainer program to maintain the culturally adapted SCP’s sustainability and broaden its reach and impact.

## Data Availability

The dataset used in the current study is available from the corresponding author upon request.
